# Intussusception Initially Diagnosed as a Brief Resolved Unexplained Event (BRUE)

**DOI:** 10.7759/cureus.39054

**Published:** 2023-05-15

**Authors:** Yukari Atsumi, Yoshiki Kusama, Sadahiro Fukui, Katsunori Kamimura

**Affiliations:** 1 Pediatrics, Amagasaki General Medical Center (AGMC), Hyogo, JPN; 2 Pediatrics, Hyogo Prefectural Amagasaki General Medical Center, Amagasaki, JPN; 3 Pediatrics, Kyoto University Hospital, Kyoto, JPN

**Keywords:** lower risk, alte not brue, intussusception, apparent life-threatening event, brief resolved unexplained event

## Abstract

Brief resolved unexplained event (BRUE) are transient and worrying episodes observed in infants and are characterized by changes in skin color, breathing, muscle tone, and/or responsiveness. We describe the case of a female infant who was initially diagnosed with BRUE but was later determined to have intussusception. She presented to our emergency department with a transient pallor and a single episode of vomiting that resolved before her visit. Physicians did not detect any abnormalities on physical or laboratory examinations, so she was diagnosed with BRUE and discharged to be re-evaluated the next day. After returning home, she vomited several times. The patient revisited our hospital the following day and was definitively diagnosed with intussusception using ultrasonography, which was successfully treated using fluoroscopy-guided hydrostatic reduction. This case was initially diagnosed as a BRUE; however, re-evaluation helped in identifying the proper diagnosis of intussusception. Physicians should exercise caution when diagnosing patients with BRUE. When the diagnostic criteria are not completely met, follow-up should be conducted, assuming that the patient has a potentially serious condition.

## Introduction

In 2016, the American Academy of Pediatrics proposed replacing the term “apparent life-threatening event” (ALTE) with “brief resolved unexplained event” (BRUE) [1]. ALTE was originally developed as a term in 1986 to replace “near-miss sudden infant death syndrome” and involved the use of various examinations and in-hospital observation to exclude serious conditions from the differential diagnoses [1]. However, serious conditions are rarely found, despite many unnecessary tests and hospitalizations [2]. By contrast, patients defined as lower risk under the BRUE guidelines require minimal additional evaluations and observations, leading to a reduction in tests and hospitalizations [3]. Despite these benefits, the complete replacement of ALTE with BRUE can be difficult, and pitfalls may exist in the process [4]. Herein, we report a case of intussusception initially diagnosed as a lower-risk BRUE with a pallor of the face at presentation. Our aim was to remind physicians of the importance of exercising caution when diagnosing BRUE.

## Case presentation

A four-month-old infant presented to our hospital with acute pallor. She had no symptoms prior to 11 p.m., when she was fed. On the same day, she suddenly began crying, swinging her limbs and head. After experiencing these symptoms, her mother noticed that her face had become pale. All symptoms improved within one minute, and the patient vomited once after the event. The patient was transported to our hospital by ambulance. She had no relevant medical history and was not taking any medication.

The physical examination and blood test results were unremarkable; the patient’s vital signs were: oxygen saturation of 97% in room air, a heart rate of 147 beats per minute, and a temperature of 36.7℃. Although the physician suspected the possibility of myocarditis, the function of the heart was found to be normal. The patient demonstrated no symptoms during follow-up observations for several hours and was able to drink milk in the emergency outpatient department. The physician confirmed the patient’s medical history as well as the results of the physical and laboratory examinations and did not detect any abnormalities. Thus, the patient was diagnosed with low-risk BRUE. She was then discharged and referred to our hospital the following morning.

**Figure 1:**
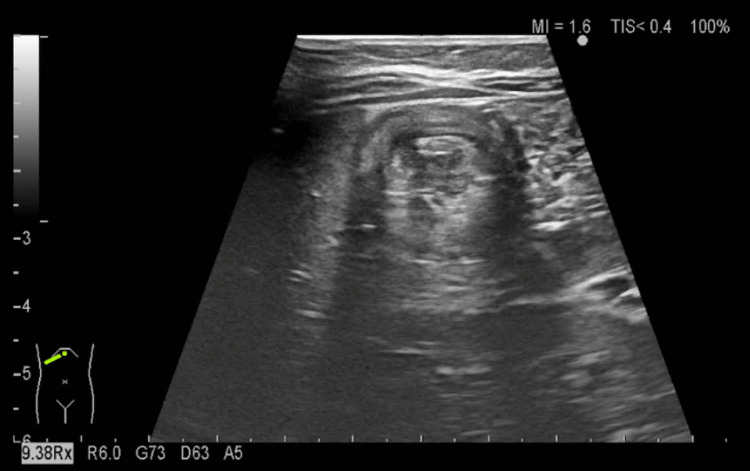
Abdominal ultrasonography Abdominal ultrasonography shows a “target sign” in the patient’s hepatic region. No echo patterns are observed in the surrounding areas

After returning home, the patient did not experience any more episodes of pallor, the chief complaint on the first visit, but did vomit non-bilious emesis repeatedly during the night. The next day, she revisited our hospital following the physician’s instructions, and an abdominal ultrasound was performed. A characteristic “target sign” was observed in her hepatic region (Figure 1), which led to a diagnosis of intussusception. The patient was then admitted for immediate treatment.

**Figure 2:**
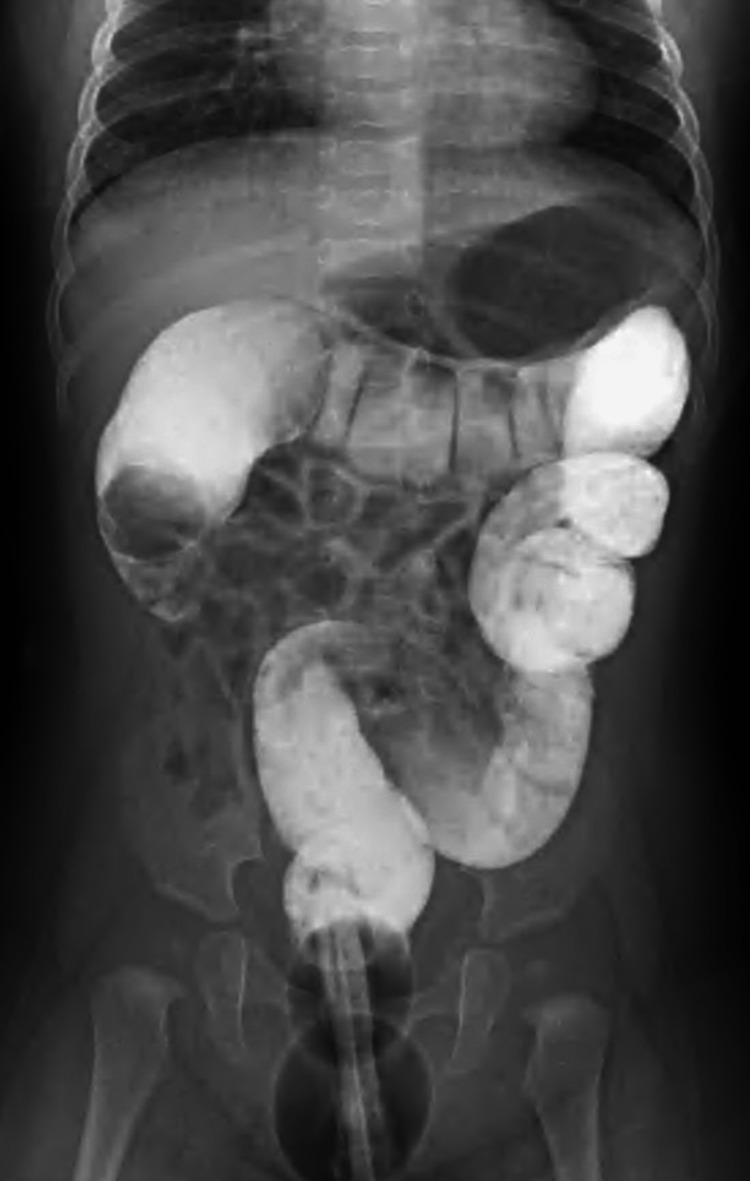
Fluoroscopy Fluoroscopy shows a “coiled-spring sign” in the hepatic hilar region.

Ultrasound- and fluoroscopy-guided hydrostatic reduction was performed using a water-soluble contrast agent, Gastrografin, suspended at a height of 90 cm. A “coiled-spring sign” was seen in the hepatic hilar region (Figure 2).

The intussusception was not resolved after the first reduction attempt (3 min) but was successfully resolved after the second. Although the patient was observed crying at night after the reduction, an abdominal ultrasound revealed no evidence of recurrence. Oral intake was resumed the day after the reduction, and the resolution of the alimentary tract obstruction was confirmed. Accordingly, we judged the intussusception to be completely reduced. The patient was discharged on the third day of hospitalization. At the time of writing this report, approximately six months after admission, the patient had not experienced any recurrence of symptoms.

## Discussion

The classical triad of intussusception includes vomiting, bloody stools, and intermittent abdominal pain (manifesting as intermittent crying in infants). Intermittent pain and bloody stools are not usually observed immediately after disease onset. Therefore, infants with intussusception may only present with vomiting. Diagnosing intussusception can be challenging since vomiting is a non-specific symptom, and its presence alone may not indicate the condition. However, close observation can help prevent a missed diagnosis. In this case, the physician in the emergency department suspected a severe underlying condition due to the transient pallor but did not focus on the vomiting as it was non-specific. We considered the possibility that the pallor was caused by a vasovagal reflex due to the pain of intussusception, as evidenced by the strong crying preceding the symptoms. While neurological signs and symptoms of intussusception, such as lethargy and hypotonia, are typically persistent [5,6], in this case, the absence of repeated crying during the transient pallor made the physician less likely to suspect abdominal pain as the cause. Although the patient did not experience recurrent pain episodes during the three hours of observation in the emergency department, re-evaluation was still necessary as intussusception remained a differential diagnosis. The patient later presented with repeated isolated episodes of vomiting, prompting the physician to perform an abdominal ultrasound at the follow-up visit, which ultimately led to a diagnosis of intussusception. Therefore, physicians should be vigilant and consider the possibility of intussusception even after a low-risk diagnosis such as BRUE, especially when symptoms persist or recur.

BRUE is defined as follows [1,3]: a sudden, brief, and now resolved episode that occurs in an infant aged < 1 year and involves cyanosis or pallor, absent, decreased, or irregular breathing, a marked change in muscle tone, and/or an altered level of responsiveness. Furthermore, BRUE should not be diagnosed for events that can be explained by a proper medical history and physical examination. BRUE is divided into two risk categories, higher and lower, based on criteria such as age and event duration [1]. As lower-risk patients are unlikely to have a recurrent event or serious disorder, physicians can omit various tests to exclude life-threatening conditions or hospitalization for observation. Patients identified as having lower-risk BRUE have good neurological outcomes and mortality rates similar to those of the general infant population, suggesting that the BRUE criteria are useful for accurately detecting lower-risk patients [1,7]. Nevertheless, many cases do not fit completely into the BRUE classification, and it is also suggested that “ALTE not BRUE” cases-patients who fulfill the criteria for ALTE but not BRUE-require careful evaluation and hospitalization [3].

The BRUE guidelines list vomiting as an exclusion criterion [1]. Therefore, in our patient's case, the pre-visit vomiting episode led to a diagnosis of "ALTE, not BRUE," which required evaluations to exclude potentially serious and urgent conditions. While the patient was initially diagnosed with a lower-risk BRUE, the physician noted the unusual occurrence of vomiting in the course of BRUE and planned a careful follow-up, which eventually led to a diagnosis of intussusception. It is important to understand that BRUE is a diagnosis based solely on symptoms and does not require a determination of the underlying etiology. Although the etiologic diagnosis is always important, aggressive etiologic evaluation is permitted to be omitted in lower-risk BRUE because data showed that exhaustive interventions for BRUE lead to an unnecessary waste of medical resources [3]. However, to avoid missing potentially dangerous diseases, the diagnosis of BRUE should be given carefully and strictly. Physicians should be aware of these exclusion criteria and consider all possible diagnoses when evaluating a patient with a suspected BRUE, even if the patient initially meets the inclusion criteria. Vigilance in considering all possible diagnoses is necessary to ensure appropriate and timely management of the patient's condition.

## Conclusions

BRUE is a useful term that allows physicians to categorize patients as "safe to discharge," but physicians need to have a clear understanding of the exact definitions and exclusion criteria. In cases where the exclusion criteria apply; or the BRUE presentation is atypical, physicians must remain vigilant and consider the possibility of a serious underlying condition. It is essential to consider all possible diagnoses to ensure appropriate and timely management of the patient's condition.
